# Outer Membrane Vesicles from *Brucella abortus* Promote Bacterial Internalization by Human Monocytes and Modulate Their Innate Immune Response

**DOI:** 10.1371/journal.pone.0050214

**Published:** 2012-11-26

**Authors:** Cora N. Pollak, M. Victoria Delpino, Carlos A. Fossati, Pablo C. Baldi

**Affiliations:** Instituto de Estudios de la Inmunidad Humoral (IDEHU), Facultad de Farmacia y Bioquímica, Universidad de Buenos Aires, Buenos Aires, Argentina; Federal University of Minas Gerais, Brazil

## Abstract

Outer membrane vesicles (OMVs) released by some Gram-negative bacteria have been shown to exert immunomodulatory effects that favor the establishment of the infection. The aim of the present study was to assess the interaction of OMVs from *Brucella abortus* with human epithelial cells (HeLa) and monocytes (THP-1), and the potential immunomodulatory effects they may exert. Using confocal microscopy and flow cytometry, FITC-labeled OMVs were shown to be internalized by both cell types. Internalization was shown to be partially mediated by clathrin-mediated endocytosis. Pretreatment of THP-1 cells with *Brucella* OMVs inhibited some cytokine responses (TNF-α and IL-8) to *E. coli* LPS, Pam3Cys or flagellin (TLR4, TLR2 and TLR5 agonists, respectively). Similarly, pretreatment with *Brucella* OMVs inhibited the cytokine response of THP-1 cells to *B. abortus* infection. Treatment of THP-1 cells with OMVs during IFN-γ stimulation reduced significantly the inducing effect of this cytokine on MHC-II expression. OMVs induced a dose-dependent increase of ICAM-1 expression on THP-1 cells and an increased adhesion of these cells to human endothelial cells. The addition of OMVs to THP-1 cultures before the incubation with live *B. abortus* resulted in increased numbers of adhered and internalized bacteria as compared to cells not treated with OMVs. Overall, these results suggest that OMVs from *B. abortus* exert cellular effects that promote the internalization of these bacteria by human monocytes, but also downregulate the innate immune response of these cells to *Brucella* infection. These effects may favor the persistence of *Brucella* within host cells.

## Introduction

Bacterial pathogens have developed numerous strategies to deliver virulence factors to the eukaryotic host cells with which they interact. Such delivery can be accomplished by either contact-dependent translocation, which mediates the direct transport of virulence factors to the host cell cytosol, or by indirect presentation, which involves the secretion of toxins and proteases to the extracellular environment for subsequent association with the host cells [1]. The specialized bacterial secretion systems, known as type I to type VII secretion systems [2]–[4], usually deliver a more or less restricted set of virulence factors whose dependency on a specific secretion system is determined by structural or amino acid sequence determinants. It has been increasingly shown that, in addition to these systems, some bacteria may use the release of outer membrane vesicles (OMVs, also known as blebs) as a mechanism for the delivery of virulence factors to host cells [5]. OMVs are closed spheroid vesicles between 10 and 300 nm in diameter that are released by Gram-negative bacteria in all growth phases [5], [6]. These vesicles are produced by budding of the outer membrane, with closure of the evaginated membrane portion at the time of release. This process results in a vesicle containing mostly outer membrane molecules with some periplasmic components inside [7]. OMVs production has been observed not only in bacteria growing in culture media but also in those growing in biofilms [8], intracellularly during *in vitro* infections [9], and even in tissues of infected patients or animals. The release of OMVs from rapidly growing meningococci was observed in a plasma sample of a young man with fatal meningococcal septicemia [10]. *Moraxella catarrhalis* present in a nasal discharge sample of a patient with sinusitis was also shown to produce OMVs [11]. Secretion of OMVs from *Acinetobacter baumannii* was detected in lung tissue of mice infected intratracheally with this bacterium [12]. Overall, these studies reveal the *in vivo* production of OMVs by different bacteria.

The role of OMVs in virulence relies in their capacity to mediate the transport of bacterial components, including virulence factors, to the interior of eukaryotic cells [5]. The mechanism for such intracellular delivery usually implies adherence of OMVs to the host cell followed by internalization. In the case of OMVs from enterotoxigenic *Escherichia coli*, *Pseudomonas aeruginosa* and *Porphyromonas gingivalis* it has been shown that vesicles associate to the lipid rafts of the host cell membrane before internalization [13]–[15]. In these and other cases it has been possible to detect the release of OMVs-associated factors inside the eukaryotic cells that have internalized the vesicles. Virulence factors detected in bacterial OMVs include adhesins, proteases (e.g., gingipains from *P. gingivalis*), and toxins (e.g., VacA from *Helicobacter pylori*) [6]. OMVs produce several effects on cells, including not only those related to the action of toxins and proteases but also immunomodulatory effects. After endocytic uptake of OMVs from *P. gingivalis* by epithelial cells, OMVs-associated gingipains degrade cellular functional molecules, including the transferrin receptor, resulting in cellular impairment [16]. Others studies have shown that *P. gingivalis* OMVs mediate CD14 degradation in human macrophages [17] and inhibition of the IFN-gamma-induced synthesis of MHC II molecules in endothelial cells [18]. Another immunomodulatory mechanism has been reported for OMVs from *Actinobacillus actinomycetemcomitans*, which contain a leukotoxin that kills human polymorphonuclear leukocytes and monocytes [19].

Previous studies have shown that smooth and rough strains of *Brucella* spontaneously release OMVs that contain outer membrane proteins, LPS and other bacterial components [20], [21]. While these OMVs were initially characterized by chemical and immunochemical methods, a proteomic analysis performed more recently [21] revealed that such vesicles contain several factors known or presumed to be related to the virulence of the bacterium, including the outer membrane proteins Omp16, Omp19, Omp25 and Omp31. It has been shown that Omp16 and Omp19 are lipoproteins that modulate MHC II expression in monocytes [22]. On the other hand, Omp25 has been linked to the ability of *Brucella* to modulate TNF-α secretion in human macrophages [23]. Therefore, it can be speculated that OMVs from *Brucella* may mediate the transfer of virulence factors to the host cell to generate immunomodulation or other effects that may favor the survival of the pathogen within cells. To our knowledge, the interaction of *Brucella* OMVs with mammalian cells and the potential immunological consequences of such interaction have not been studied. The evaluation of these phenomena was the goal of the present study.

## Materials and Methods

### Isolation of Outer Membrane Vesicles and Fluorescent Labeling

OMVs were obtained by a modification of the original method of Gamazo and Moriyón [20]. *B. abortus* 2308 were grown overnight in tryptic soy broth (TSB), harvested by centrifugation, and washed twice in phosphate-buffered saline (PBS). The pellet was resuspended in Gerhardt-Wilson minimal medium at an OD_600 nm_ of 0.1, cultured for 72 h and harvested by centrifugation at the early stationary phase of growth. The cell-free supernatant was passed through 0.22 µm-pore-size filters to remove the remaining bacteria. An aliquot of the filtrate was tested for the presence of viable *B. abortus* cells by plating on TSB agar. In all cases, no colonies were detected. The filtrate was ultracentrifuged at 100,000×g for 5 h at 4°C to pellet the vesicles. The supernatant was carefully removed and the pellets were resuspended in PBS. Protein concentration was measured using a bicinchoninic acid (BCA) assay (Pierce). OMVs were stored at −20°C until use. Electron microscopy studies revealed that these storage conditions did not affect OMVs morphology. For studies requiring fluorescent labeling, vesicles were incubated ON at 4°C with fluorescein isothiocyanate (FITC; Sigma–Aldrich, USA; 1∶1) and dialyzed against PBS to remove the unbound stain.

### SDS–PAGE and Immunoblotting

Purified OMVs (5 µg of proteins) were resolved by SDS-PAGE and transferred to a nitrocellulose membrane. The membranes were subjected to Western blot analysis with an anti-*Brucella* LPS monoclonal antibody prepared in our laboratory (1∶10 dilution).

### Negative Staining Electron Microscopy

OMVs obtained by ultracentrifugation were suspended in distilled, deionized H_2_0, applied to 300 mesh formvar-coated copper grids, fixed with 1% OsO_4_ for 30 min, and stained with 2% phosphotungstic acid. Preparations were examined in a transmission electron microscope (Zeiss 10).

### Cell Culture

Unless otherwise specified, all experiments were performed at 37°C in a 5% CO_2_ atmosphere. THP-1 cells were obtained from the American Type Culture Collection (Manassas, VA) and were grown and maintained in RPMI 1640 supplemented with 2 mM L-glutamine, 10% heat-inactivated fetal bovine serum, 100 U of penicillin per ml, and 100 µg of streptomycin per ml. To induce maturation, the cells were cultured in the presence of 0.05 µM 1,25-dihydroxyvitamin D_3_ (Calbiochem-Nova Biochem International, La Jolla, CA) for 48 to 72 h. HeLa cells were obtained from our institutional collection and were grown and maintained in Dulbecco's Modified Eagle Medium (DMEM) supplemented with 2 mM L-glutamine, 10% heat-inactivated fetal bovine serum, 100 U of penicillin per ml, and 100 µg of streptomycin per ml. Human dermal microvascular endothelial cells (HMEC-1) were purchased from the American Type Culture Collection and cultured in MCDB131 medium (Invitrogen, Carlsbad, CA) containing 10 ug/ml hydrocortisone, 1 ng/ml epidermal growth factor (BD Pharmingen, San Jose, CA), 10% fetal calf serum, 2 mM L-glutamine, 100 U/ml penicillin and 100 mg/ml streptomycin.

### Confocal Microscopy

To investigate the OMVs association to non phagocytic and phagocytic cells, HeLa and vitamin D3-treated THP-1 cells were seeded onto glass coverslips (4×10^5^/well), grown till confluence and labeled with the lipophilic DiD dye according to the manufacteŕs instructions (Vybrant™ Molecular Probes). DiD-labeled THP-1 (1×10^6^/ml) and DiD-labeled HeLa cells were incubated with 10 µg of FITC-OMVs for 4 h and 24 h respectively before microscopical examination. Cells were examined and imaged using an Olympus FV300 fluorescence microscope and FluoView software.

### Flow Cytometry Analysis

Vitamin D3-treated THP-1 cells (1×10^6^/ml) were cultured with FITC-labeled OMVs (10 µg OMVs protein) for up to 4 h. HeLa cells (6×10^4^/well) were cultured overnight till confluent in 24 well plates, washed once with PBS, and incubated with FITC-labeled OMVs (10 µg OMVs protein) in complete culture medium for up to 24 h. After incubation, cells were washed to remove unbound OMVs. THP-1 cells were harvested by centrifugation and HeLa cells were harvested after incubation with trypsin/EDTA. Fluorescence measurements were made using a flow cytometer (Partec, Pas III model) and Flomax software. A total of twenty thousand events were collected for each sample. Mean fluorescence intensity (MFI) values of cells incubated in the absence of OMVs were subtracted from the values of OMVs-treated cells. To determine the proportion of internalized OMVs, the fluorescence of non-internalized cell-associated OMVs was quenched by the addition of trypan blue (0.025% final concentration); fluorescence was measured before and after the addition of the stain [24].

To assess internalization mediated by lipid raft/caveolae or by clathrin- or actin mediated endocytosis cells were pretreated for 30 min with different doses of Filipin III (1, 5, 10 or 20 µg/ml), monodansylcadaverine (MDC; 50, 100, 200 or 300 µM) or Cytochalasin D (1 µg/ml), and then incubated for 1 h with FITC-OMVs (2.5 µg/ml, final concentration) in the presence of these inhibitors. In all the experiments control cells were incubated without inhibitor or with DMSO (vehicle of inhibitors) for the same period of time. Fluorescence measurements were performed as described above.

### Effect of OMVs on the Cytokine Response of Monocytes to TLR Agonists or *B. abortus* Infection

Vitamin D3-treated THP-1 cells were pre-stimulated for 4 h with OMVs (0.1, 1 or 10 µg/ml) washed twice, and incubated with LPS (100 ng/ml), Pam_3_Cys (50 ng/ml) or flagellin from *Salmonella enterica* serovar *Typhimurium* (5 ng/ml) for 24 h. Supernatants were harvested and their content of human TNF-α, IL-8, and IL-1β was measured by sandwich ELISA using paired cytokine-specific mAbs, according to the manufacturer’s instructions (BD Biosciences).

In other experiments cells were pre-stimulated with OMVs and washed as described above before being infected with *B. abortus* at a multiplicity of infection (MOI) of 100 for 1 h in standard medium containing no antibiotics. Cells were extensively washed with RPMI and were subsequently incubated for 24 h in culture medium containing 100 µg/ml gentamicin and 50 µg/ml streptomycin to kill extracellular bacteria. Culture supernatants were harvested and analyzed for cytokines content as described above.

### Effect of OMVs on MHC-II Expression

Vitamin D3-treated THP-1 cells at a concentration of 0.5 × 10^6^ cells/ml were incubated for 48 h in round-bottom polypropylene tubes with or without 150 U/ml of recombinant human gamma-interferon (IFN-γ, Endogen) in the absence or presence of OMVs (0.1, 1 or 10 µg/ml). At the end of culture, cells were washed and blocked with human serum for 15 min, and then incubated with fluorescein isothiocyanate-labeled anti-human HLA-DR monoclonal antibody (MAb) (BD Pharmingen), or isotype-matched control antibody (Ab) for 30 min on ice. The cells were then washed and analyzed in a flow cytometer (Partec, Pas III model), and results were processed with Flomax software. A total of twenty thousand events were collected for each sample. The results were expressed as mean fluorescence intensities (arithmetic means ± standard errors of the means).

### Analysis of Adhesion Molecules Expression

THP-1 cells (1×10^6^) were cultured with the addition of OMVs (10 µg of OMVs, protein basis) for 4, 12, 16 and 24 h. At the end of each culture, cells were washed with PBS-BSA 1% and blocked with human serum for 15 min on ice, incubated for 30 min with mouse monoclonal antibodies against human CD54 (ICAM-1) or CD106 (VCAM-1) or with isotype-matched control antibodies (all from BD Pharmingen, San Jose, CA). Cells were then washed, and incubated with a FITC-labeled goat anti-mouse antibody, fixed with 4% paraformaldehyde, and analyzed with a flow cytometer (Partec, Pas III model) equipped with Flomax software. The results were expressed as mean fluorescence intensity (MFI).

### Cell-binding Assay

The ability of *B. abortus* OMVs to stimulate the adhesion of monocytes to endothelial cells was analyzed using the human monocytic cell line THP-1 and the human endothelial cell line HMEC-1. The latter were seeded in 96-well plates at a density of 1×10^4^ cells per well, cultured to confluence and then stimulated with 25 ng/ml TNF-α for 6 h. In parallel, THP-1 cells were stimulated with increasing concentrations of OMVs (0.1, 1 and 10 µg/ml) for 24 h in culture medium, washed, labeled with calcein acetoxymethylester fluorescent dye (BD Biosciences, 5µM final concentration) for 30 min at 37°C, washed, resuspended in sterile PBS, and added (1×10^5^ cells per well) to TNF-α stimulated HMEC-1 for 1 h at 37°C in a humidified atmosphere with 5% CO_2_. As controls, non-stimulated THP-1 cells were added to TNF-α stimulated HMEC-1 cells or to non-stimulated HMEC-1 cells. After incubation, non-adherent THP-1 cells were carefully washed off with PBS. Cell fluorescence intensity was measured in a fluorescence plate reader (VICTOR™, PerkinElmer) at excitation/emission wavelengths of 494/517 nm, and cell adhesion was expressed as a percentage of the total number of THP-1 cells added, according to the equation: adhesion rate = fluorescence intensity of adherent THP-1 cells/fluorescence intensity of total applied THP-1 cells×100%. All assays were performed in triplicate and the binding of non-stimulated THP-1 cells to TNF-α stimulated HMEC-1 cells was used as an indicator of basal cell adherence.

Additional experiments were carried out to determine whether OMVs can stimulate the adhesive properties of endothelial cells. To this end, HMEC-1 cells were incubated with 10 µg/ml of OMVs for 24 h in culture medium and washed before the addition of either resting or OMVs-activated THP-1 cells (10 µg/ml dose). Coincubation, washing and reading were performed as described above.

### Effect of Preincubation or Coincubation with OMVs on the Adhesion and Internalization of *Brucella* in Human Monocytes

THP-1 cells (0.5×10^6^) were preincubated or not for 30 min with different doses of OMVs (0.1, 1 or 10 µg/ml), and were infected with *B. abortus* at an MOI of 50 for 1 h in standard medium containing no antibiotics. Cells were extensively washed with RPMI and were subsequently incubated for 1 h in standard medium, or for 1 h or 24 h in the same medium containing 100 µg/ml gentamicin and 50 µg/ml streptomycin to kill extracellular bacteria. Cells were washed with sterile PBS, lysed with 0.1% (vol/vol) Triton X-100 in H_2_O, and serial dilutions of lysates were rapidly plated onto tryptose soy agar plates to enumerate colony-forming units (CFU). The number of adherent extracellular bacteria was calculated by subtracting the number of intracellular bacteria from the total count obtained in the absence of antibiotics.

A similar approach was used to evaluate the effect of the presence of OMVs during the infection. THP-1 cells were infected for 1 h with *B. abortus* at a MOI of 50 as described above in the presence of different doses of OMVs (0.1, 1 or 10 µg/ml). At the end of the infection period cells were processed for determining the numbers of adherent and intracellular bacteria as described above.

### Effect of Preincubation or Coincubation with OMVs on the Adhesion and Phagocytosis of Latex Beads by Human Monocytes

THP-1 cells (0.5×10^6^) were preincubated or not for 30 min with different doses of OMVs (0.1, 1 or 10 µg/ml) before incubation with FITC-labeled latex beads (2.0 µm mean particle size, Sigma) for 30 min at 37°C in a 5% CO_2_ atmosphere. Cells were extensively washed and were analyzed by flow cytometry as detailed above, with or without the addition of trypan blue to quench the fluorescence of non-internalized cell-associated beads. In a separate experiment, OMVs and latex beads were added concomitantly to THP-1 cells and the culture was incubated for 1 hour before flow cytometry analysis.

### Statistical Analysis

Statistical analyses were performed by one-way ANOVA followed by either Dunnetts test (comparison with control group) or Tukey’s test (comparison between several groups), using the GraphPad Prism 4.0 software.

## Results

### Isolation of *Brucella abortus* OMVs

**Figure 1:**
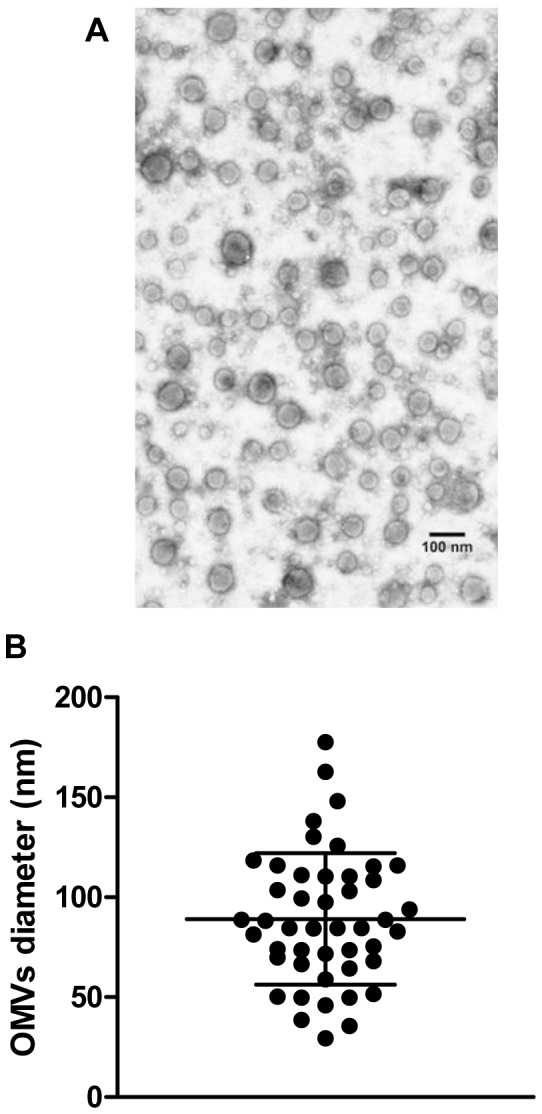
Isolation and analysis of *B. abortus* outer membrane vesicles (OMVs). (A) Electron micrograph of the OMVs suspension used in this study. (B) Size distribution of OMVs. Horizontal lines represent mean and standard deviation values.

According to the observation of the OMVs through electron microscopy (Fig. 1A), the diameters of isolated OMVs ranged from 30 to 178 nm (median: 85 nm; mean: 89 nm; standard deviation: 33 nm) (Fig. 1B). The absence of bacterial debris confirmed the purity of the OMVs fraction. In addition, an immunoblot analysis of purified OMVs with a specific monoclonal antibody confirmed the presence of *Brucella* LPS in the vesicles (not shown).

### *B. abortus* OMVs are Internalized by Phagocytic and Non-phagocytic Cells

**Figure 2:**
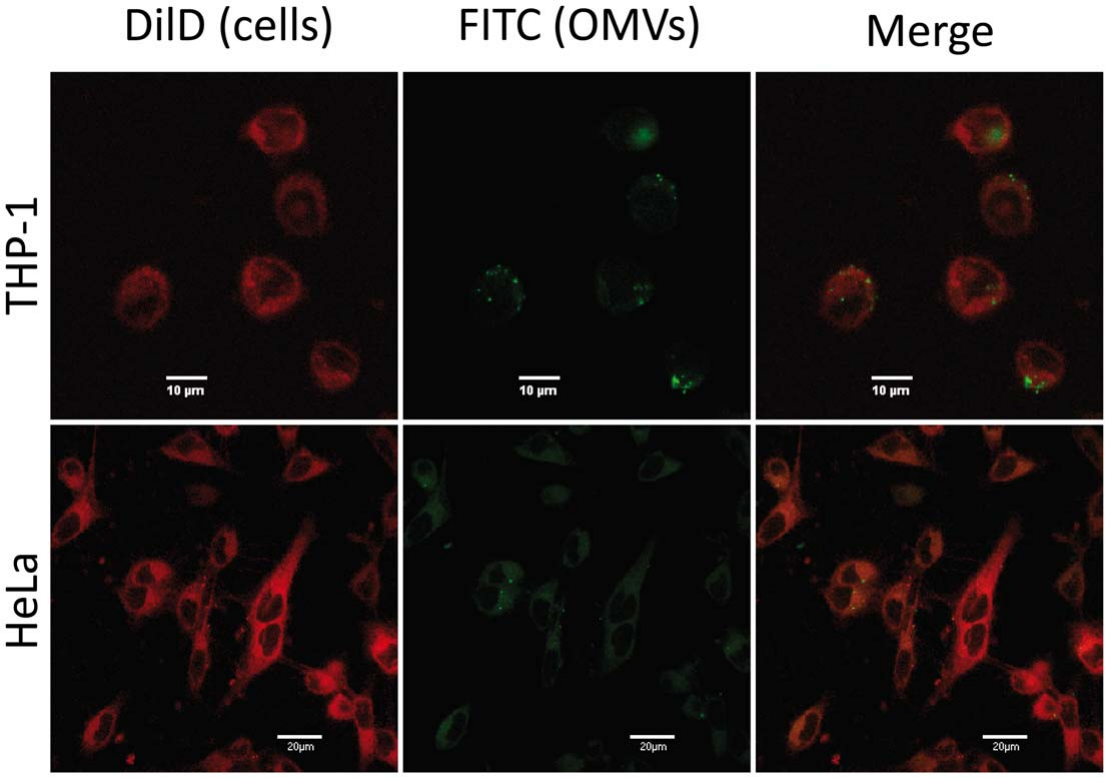
OMVs are internalized by THP-1 and HeLa cells. FITC-labeled-OMVs were incubated with DiD-stained THP-1 cells for 4 h at 37°C (upper panels) or with DiD-stained HeLa cells for 24 h (lower panels). After incubation, cells were washed, fixed in 4% paraformaldehyde, and visualized by confocal microscopy. Left panels show DiD fluorescence (cells), middle panels show FITC fluorescence (OMVs), and right panels show merged images.

The human epithelial cell line HeLa and the monocytic cell line THP-1 were stained with the membrane-specific stain DiD before incubating them with FITC-labeled OMVs from *B. abortus*. After washing and fixing, cells were analyzed by confocal microscopy. As shown in Figure 2, some OMVs adhered to the membrane of epithelial or monocytic cells while others were detected inside the cells.

**Figure 3:**
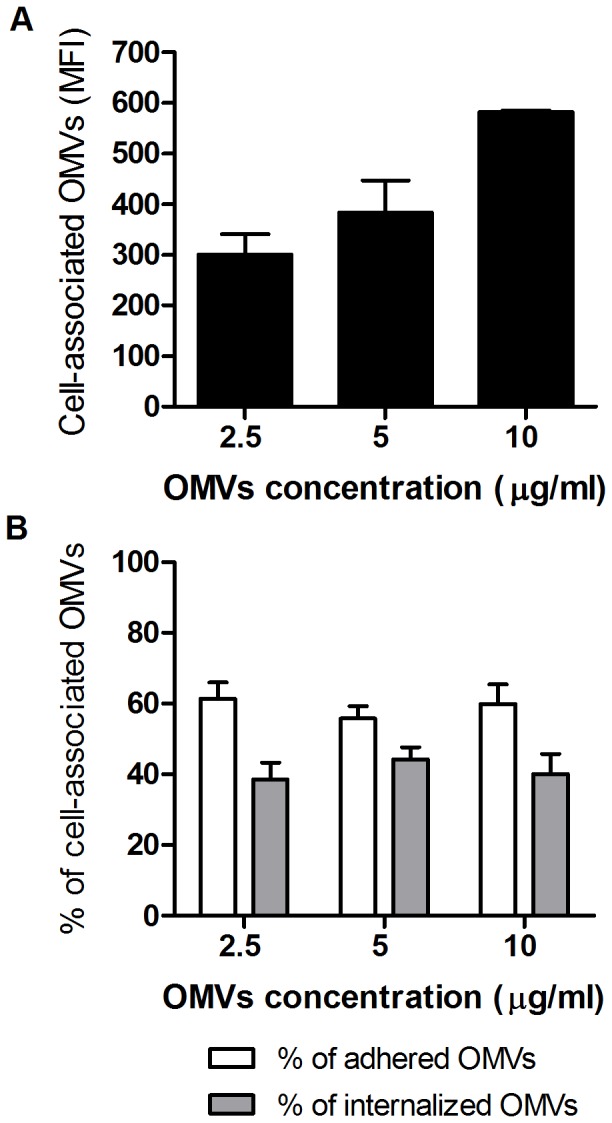
Flow cytometry assessment of the association of FITC-labeled OMVs with THP-1 cells. Cells were incubated for 4 h with 2.5 to 10 µg/ml of labeled OMVs. A) Dose-dependent OMVs uptake by THP-1 cells expressed as mean fluorescence intensity (MFI). B) Proportion of adhered and internalized OMVs for the different OMVs doses tested. Fluorescence was measured before and after the addition of trypan blue (used to quench extracellular fluorescence) in order to estimate the proportion of total cell-associated OMVs and intracellular OMVs, respectively. Data shown are from one of three independent experiments done in duplicate, which yielded similar results.

To obtain an estimation of the proportion of cells with internalized OMVs, HeLa and THP-1 cells were incubated with increasing concentrations of FITC-labeled OMVs, washed to eliminate unbound OMVs, and analyzed by flow cytometry. To determine the proportion of internalized OMVs, the fluorescence of non-internalized cell-associated OMVs was quenched by the addition of trypan blue (0.025% final concentration); fluorescence was measured before (total associated OMVs) and after (intracellular OMVs) the addition of the stain. As shown in Figure 3 increasing concentrations of OMVs resulted in a linear increase in the number of total cell-associated vesicles (Fig. 3A), but the proportions of adhered and internalized OMVs were similar along the full range of concentrations, with a preponderance of adhered vesicles (Fig. 3B). Similar results were obtained for HeLa cells (not shown). In the experiments with THP-1 cells the percentage of cells with associated OMVs (both intracellular and extracellular) ranged from 95% to 98%.

### OMVs Uptake by Monocytes Depends Mainly on Clathrin-mediated Endocytosis

**Figure 4:**
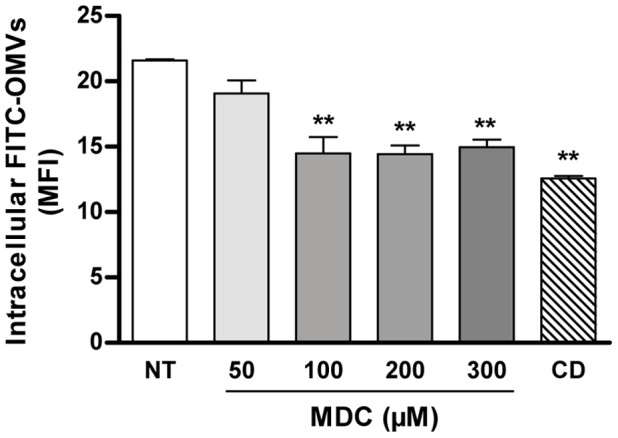
Effect of endocytosis inhibitors on the internalization of *Brucella* OMVs in monocytic THP-1 cells. Cells were pretreated with different doses of MDC or with 1 µg/ml Cytochalasin D for 30 min before the addition of FITC-labeled OMVs (1 µg/ml, final concentration). Inhibitors were maintained during incubation with OMVs (1 h). The total number of cells with associated OMVs was determined by flow cytometry as indicated in Figure 3. Data shown are from one of three independent experiments done in duplicate, which yielded similar results. Asterisks indicate significantly differences between cells pretreated with inhibitors and untreated cells (**,p<0.01; ANOVA followed by Dunnett’s test). NT: not treated.

Three major endocytic pathways have been described in mammalian cells, including a) clathrin-mediated endocytosis, characteristic of receptor-mediated endocytosis, b) invagination of cholesterol-enriched microdomains within the plasma membrane known as lipid rafts or caveolae, and c) formation of large F-actin coated vacuoles that serve to uptake either solid particles or liquid from the extracellular space (phagocytosis and macropinocytosis, respectively) [25]. To examine which pathways may be involved in the uptake of *Brucella* OMVs by monocytes, THP-1 cells were pretreated with Filipin III, which disrupts lipid rafts but does not affect phagocytosis and clathrin-mediated endocytosis, with monodansylcadaverine (MDC) which is a relatively specific blocker of clathrin-mediated internalization, or with cytochalasin D, which blocks actin polymerization and is considered a global and nonselective inhibitor of all internalization pathways [25]. As shown in Fig. 4 cytochalasin D inhibited significantly (by 42%) the internalization of OMVs in THP-1 cells. Internalization was also inhibited by different doses of MDC (by 12% with 50 µM, and by 33% with 100 µM or higher doses). In contrast, no significant inhibition of vesicles uptake was observed with different concentrations of Filipin III (not shown). These results suggest that clathrin-mediated endocytosis is the main mechanism involved in the internalization of *Brucella* OMVs into human monocytes, although an additional, albeit minor contribution of other pathways cannot be ruled out.

### OMVs Modulate the Cytokine Response of Host Cells to TLR Agonists

**Figure 5:**
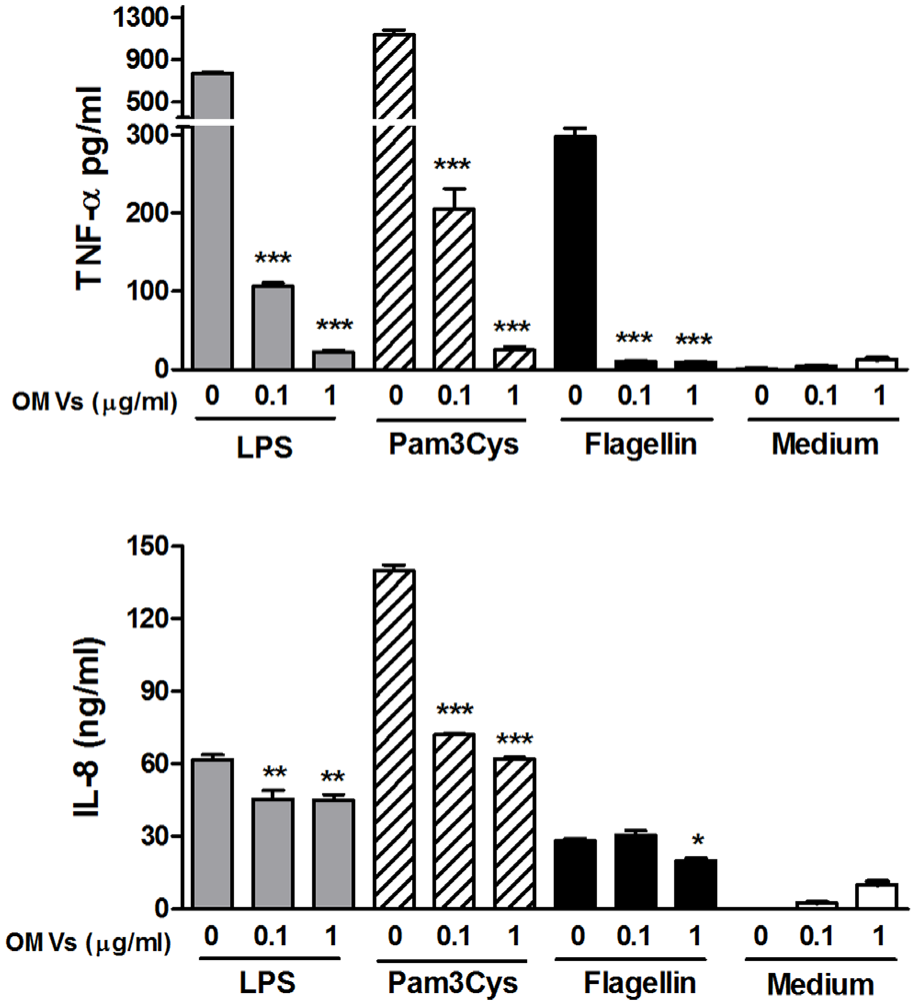
Pretreatment with *Brucella* OMVs inhibits the cytokine response of THP-1 cells to TLR agonists. THP-1 cells were incubated or not with OMVs (0.1 or 1.0 µg/ml) for 4 h before stimulation for 24 h with LPS from *E. coli*, Pam3cys or flagellin from *Salmonella enterica* serovar Typhimurium (TLR4, TLR2 and TLR5 agonists, respectively). Cytokines were measured in culture supernatants by commercial ELISAs. Data shown are from one of three independent experiments done in duplicate, which yielded similar results. Asterisks indicate significantly different responses to a particular TLR agonist between cells pretreated or not with OMVs (*, p<0.05; **, p<0.01; ***, p<0.001, ANOVA followed by Dunnett’s test.

As mentioned, *B. abortus* OMVs contain Omp25, which has been implicated in the ability of *Brucella* to downregulate LPS-induced TNF-α secretion in human monocytes [23]. Therefore, experiments were performed to assess whether preincubation with *B. abortus* OMVs can modulate cytokine responses of human monocytes in response to different TLR agonists. To this end, THP-1 cells preincubated (or not) with OMVs were incubated for 24 h with LPS from *E. coli*, Pam_3_Cys or flagellin (TLR4, TLR2 and TLR5, agonists respectively) and cytokines were measured in culture supernatants by commercial ELISAs. As shown in Figure 5A, preincubation of cells with 1 µg/ml of OMVs inhibited the TNF-α response to LPS, Pam3Cys and flagellin by 97%, 98% and 97%, respectively, and in the case of IL-8 (Figure 5B) inhibition percentages were 27%, 56% and 29%, respectively. Inhibition was also observed with the lower dose of OMVs tested (0.1 µg/ml), which inhibited the TNF-α response to LPS, Pam3Cys and flagellin by 86%, 82% and 96%, respectively (Figure 5A), and the IL-8 response to LPS and Pam3Cys by 26% and 48%, respectively (Figure 5B) (response to flagellin was not inhibited). Preincubation of THP-1 cells with 10 µg/ml of *Brucella* OMVs (not shown) inhibited by 95%, 97% and 93% the TNF-α secretion in response to LPS, Pam_3_Cys and flagellin, respectively, and the IL-8 response to these agonists by 64%, 82% and 44%, respectively. In contrast, no inhibitory effect was observed on IL-1β secretion for all the OMVs doses tested (not shown).

### OMVs Modulate the Cytokine Response of Host Cells to *B. abortus* Infection

**Figure 6:**
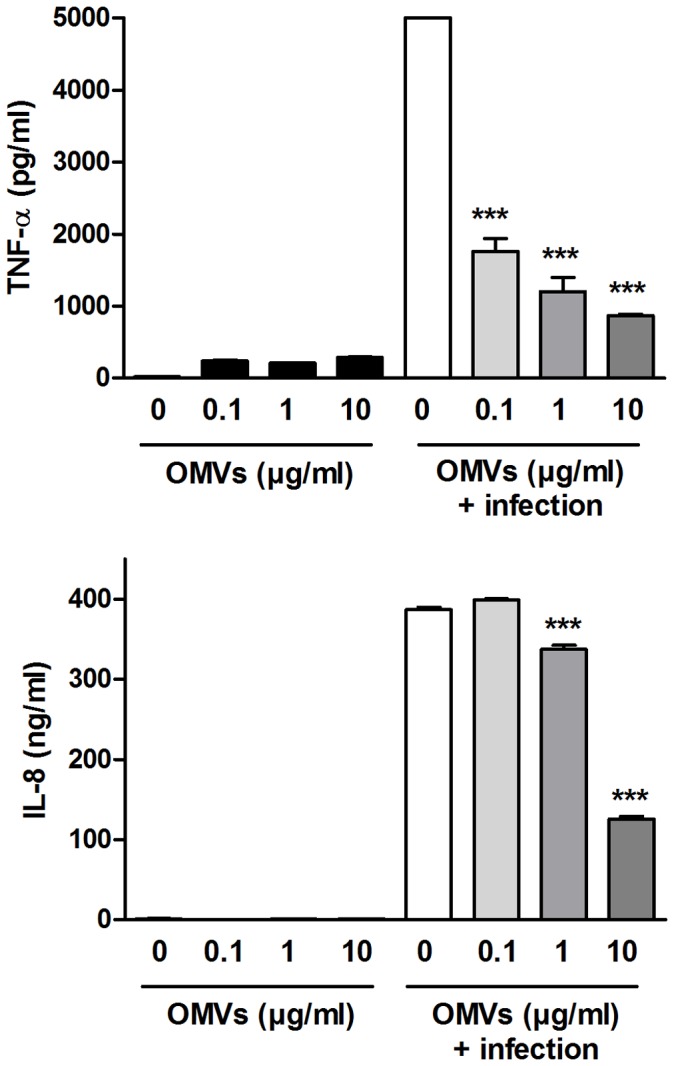
Pretreatment with *Brucella* OMVs decreases the cytokine response of THP-1 cells to *Brucella* infection. THP-1 cells were incubated or not with OMVs (0.1 to 10 µg/ml) for 4 h before infection with *B. abortus* (MOI 100) for 1 h. Cytokines were measured by commercial ELISAs in culture supernatants collected at 24 h p.i. Data shown are from one of three independent experiments done in duplicate, which yielded similar results. Asterisks indicate significantly different responses to infection between cells pretreated with OMVs and untreated cells (***, p<0.001, ANOVA followed by Tukey’s test).

The inhibitory effect of OMVs on the cytokine response of THP-1 cells to TLR agonists suggested a potential role of OMVs release as an immunomodulatory mechanism during *Brucella* infections. To test this possibility THP-1 cells were preincubated or not with different doses of OMVs before *B. abortus* infection, and the levels of TNF and IL-8 were measured in culture supernatants at 24 h p.i. As shown in Figure 6, preincubation with OMVs at 0.1 to 10 µg/ml inhibited significantly (by 65%, 76% and 83%) the production of TNF-α by THP-1 cells in response to *B. abortus* infection. Similarly, preincubation of THP-1 cells with OMVs at 1 and 10 µg/ml inhibited significantly (by 13% and 68%) the production of IL-8 in response to infection. Therefore, these results indicate that the previous interaction of OMVs with human monocytes conditions the cytokine response of these cells to a subsequent *B. abortus* infection.

### *Brucella* OMVs Inhibit the IFN-γ-induced Expression of MHC-II Molecules on Human Monocytes

**Figure 7:**
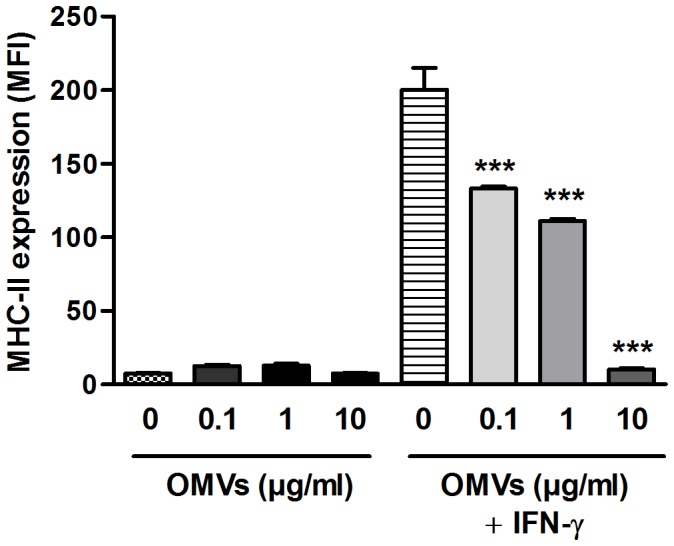
Pretreatment with *Brucella* OMVs decreases the IFN-γ-induced expression of MHC-II molecules on human monocytes. THP-1 cells were incubated for 48 h with different doses of OMVs (0.1 to 10 µg/ml) in the presence or absence of IFN-γ (150 U/ml) before evaluating MHC-II expression by flow cytometry. Data shown are from one of three independent experiments done in duplicate, which yielded similar results. Asterisks indicate significant differences in MHC-II expression between cells treated with OMVs during IFN-γ stimulation and those that only received IFN-γ stimulation (***, p<0.001, ANOVA followed by Tukey’s test).

It is well established that IFN-γ induces the expression of MHC-II molecules on a wide range of cell types, including monocytes and macrophages [26], [27]. Previous studies have shown that the presence of the lipoproteins Omp16 or Omp19 from *B. abortus* during the stimulation of human monocytes with IFN-γ results in a reduced expression of MHC-II molecules as compared to cells not treated with these lipoproteins [22]. Since *B. abortus* OMVs have been shown to contain these lipoproteins [21], experiments were carried out to determine whether treatment of human monocytes (THP-1 cells) with these OMVs during IFN-γ stimulation may also inhibit MHC-II expression. As expected IFN-γ induced a significant increase of MHC-II expression on THP-1 cells (MFI 200.2 vs. 7.67 of the unstimulated control) (Figure 7). However, the inducing effect of IFN-γ on MHC-II expression was significantly inhibited when different doses of OMVs were added together with IFN-γ. MFI values were reduced by 33%, 45% and 95% by treatment with 0.1, 1.0 and 10 µg/ml of OMVs, respectively.

### Stimulation with OMVs Induces the Expression of Adhesion Molecules

**Figure 8:**
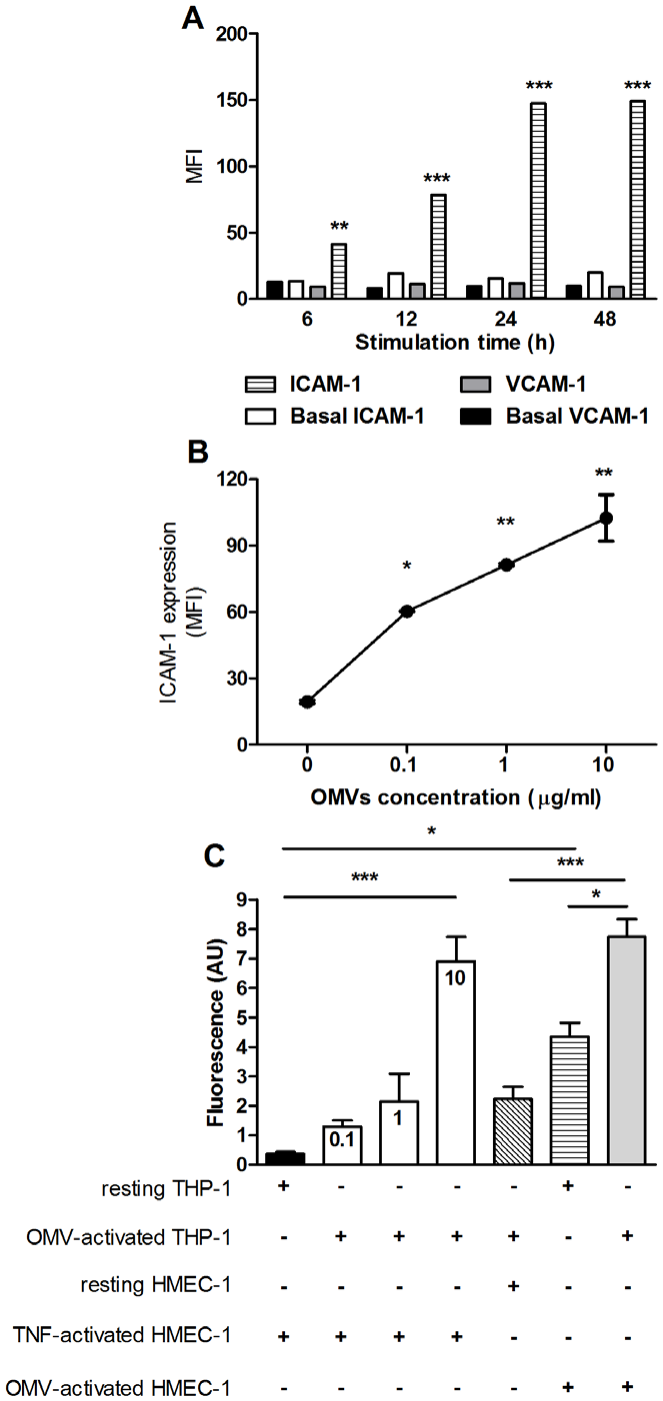
Stimulation with OMVs increases ICAM-1 expression and adhesion of human monocytes to endothelial cells. (A) THP-1 cells were incubated with OMVs (10 µg/ml) for different periods and the surface expression of adhesion molecules (ICAM-1 and VCAM-1) was determined by flow cytometry. Asterisks indicate significant differences for each time in expression between cells treated or not with OMVs (**, p<0.01, ***, p<0.001, ANOVA followed by Tukey’s test). (B) THP-1 cells were incubated with different doses of OMVs for 24 h and ICAM-1 expression was measured by flow cytometry. Asterisks indicate significant differences between cells treated with OMVs and untreated cells (basal) (*, p<0.05, **, p<0.01, ANOVA followed by Dunnett’s test). (C) THP-1 cells were incubated or not with different doses of OMVs (indicated inside columns), labeled with calcein, and dispensed on a monolayer of human endothelial cells (HMEC-1) previously activated with TNF-α or incubated with OMVs, or left untreated (resting). After coincubation, non-adhered cells were eliminated and the fluorescence of adhered cells was measured. Asterisks indicate significant differences between treatments (*, p<0.05, ***, p<0.001, ANOVA followed by Tukey’s test). In all cases data shown are from one of three independent experiments done in duplicate, which yielded similar results.

As mentioned, OMVs contain a complex mixture of outer membrane and periplasmic bacterial antigens, some of which may act as stimulants of innate immune responses. One of such responses may be the increased expression of adhesion molecules that contribute to the migration of immune cells through the endothelium towards the focus of infection. To determine whether such response is induced by *Brucella* OMVs, human monocytes (THP-1 cell line) were incubated with different doses of these vesicles and the surface expression of adhesion molecules (ICAM-1 and VCAM-1) was determined by flow cytometry. As shown in Figures 8A and 8B, OMVs induced a time-dependent and dose-dependent increase of ICAM-1 expression on THP-1 cells, although they did not modify the expression levels of VCAM-1.

To determine whether the interaction of monocytes with *Brucella* OMVs results in an increased adhesion of these cells to the endothelium, THP-1 cells were incubated with different doses of OMVs, labeled with calcein, and dispensed on a monolayer of human endothelial cells (HMEC-1). After coincubation, non-adhered cells were eliminated and the fluorescence of adhered cells was measured. As shown in Figure 8C, OMVs induced a dose-dependent increase of monocyte adherence to endothelial cells, although a statistically significant increase was obtained only with the highest OMVs dose (10 µg/ml). Experiments were also carried out to determine whether OMVs can stimulate the adhesive properties of endothelial cells. As shown in Figure 8C, the adhesion of unstimulated monocytes to OMVs-stimulated HMEC-1 cells was higher than that to unstimulated HMEC-1 cells and was also higher than adhesion to TNF-activated HMEC-1 cells, although differences did not reach statistical significance in any case. Overall, these results suggest that *Brucella* OMVs stimulate the expression of adhesion molecules on the surface of both monocytes and endothelial cells, thus favoring adhesive interactions between these cell types.

### Preincubation or Coincubation with OMVs Enhances the Adhesion and Internalization of *B. abortus* in Human Monocytes

**Figure 9:**
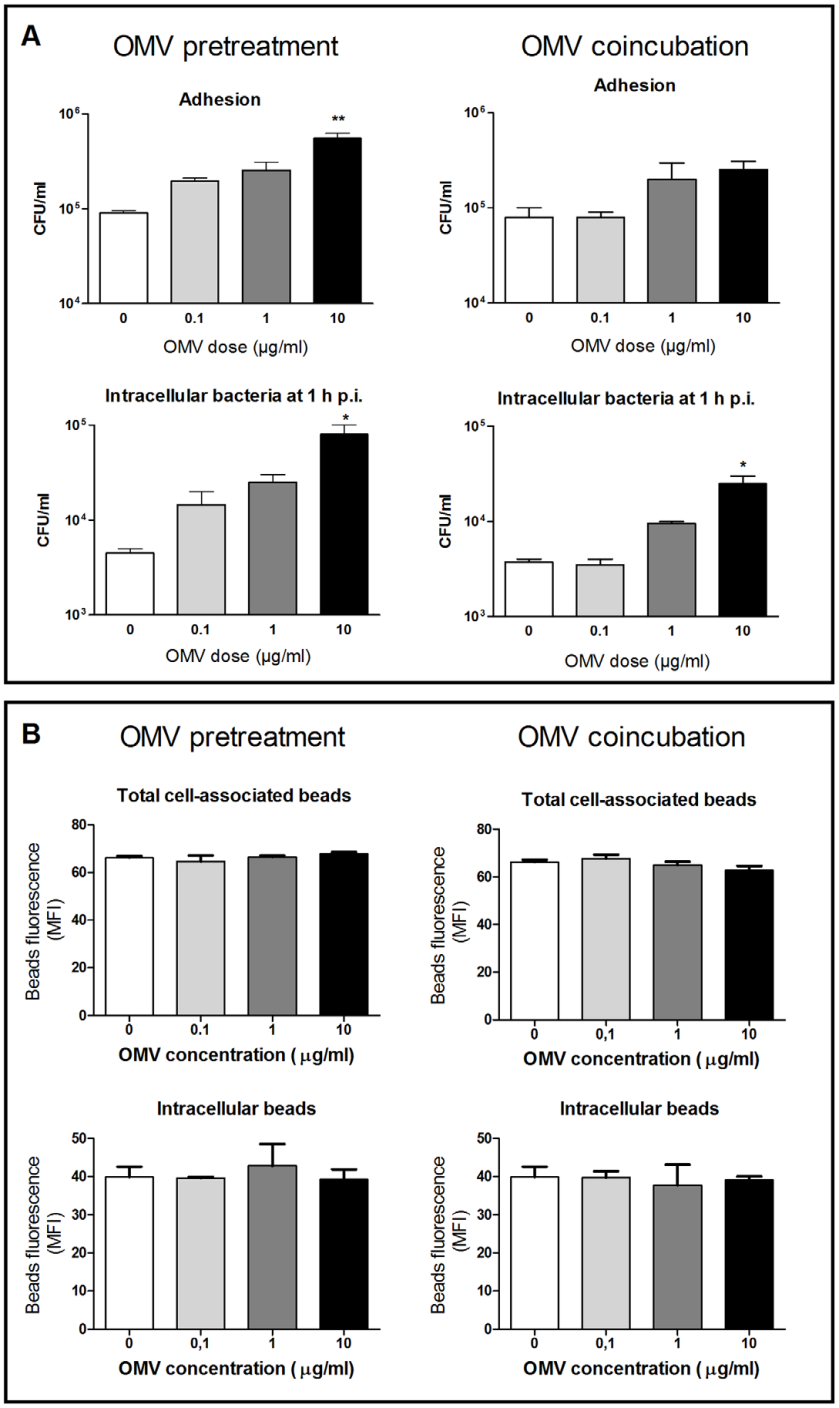
Preincubation and coincubation with OMVs enhance *Brucella* adhesion and internalization by human monocytes but do not modify the adhesion and internalization of fluorescent latex beads. (A) THP-1 cells were either preincubated with OMVs during 30 min before adding viable *B. abortus* to the cell culture, or OMVs were added together with the bacteria. After an infection period of 1 h the cells were washed and the numbers of adhered and internalized bacteria were determined. Data shown are from one of three independent experiments done in duplicate, which yielded similar results. Asterisks indicate significantly different CFU numbers between cells preincubated or coincubated with OMVs and those that were not treated with these vesicles (*, p<0.05; **, p<0.01, ANOVA followed by Dunnett’s test). (B) THP-1 cells were either preincubated with OMVs during 30 min before adding the fluorescent latex beads to the cell culture, or OMVs were added together with the beads. After an incubation period of 30 min, the cells were washed and the fluorescence of total cell-associated beads (in the absence of trypan blue) and internalized beads (in the presence of trypan blue) were determined by flow cytometry. Results are expressed as mean fluorescence intensity (MFI). Data shown are from one of three independent experiments done in duplicate, which yielded similar results.

Some of the results described above indicated that OMVs exert effects, such as TNF-α inhibition and MHC-II downregulation, which may favor *Brucella* persistence after infection. We wondered whether the previous or simultaneous interaction of OMVs with target cells may also influence on the internalization of *Brucella* by such cells. To test this possibility, THP-1 cells were either preincubated with OMVs during 30 min before adding viable *B. abortus* to the cell culture, or OMVs were added together with the bacteria. After an infection period of 1 h the cells were washed and the numbers of adhered and internalized bacteria were determined. As shown in Figure 9A, preincubation with OMVs resulted in increased numbers of adhered and internalized bacteria in THP-1 cells, and these effects reached statistical significance for the highest OMVs dose (p<0.01 and p<0.05, respectively). Coincubation with OMVs also increased the adhesion and internalization of *B. abortus* to THP-1 cells, although differences were only significant (p<0.05) for the internalization attained upon coincubation with the highest dose. These results suggest that OMVs released by *B. abortus* before or during the interaction with monocytes produce a significant increase in the internalization of the bacterium by these cells.

We hypothesized that the increased internalization of *B. abortus* by THP-1 cells preincubated or coincubated with OMVs may be due to an increased phagocytic activity of monocytes as a result of OMV treatment or an increased expression of a cellular receptor for *Brucella*. To test the first possibility, THP-1 cells were incubated with different doses of OMVs either before or during incubation with FITC-labeled latex beads (2.0 µm mean particle size). Cells were extensively washed and were analyzed by flow cytometry, with or without the addition of trypan blue to quench the fluorescence of non-internalized cell-associated beads. As shown in Figure 9B, neither preincubation nor coincubation with OMVs induced significant changes in the number of cell-associated or internalized latex beads as compared with untreated cells. These results suggest that the increase in *Brucella* uptake by THP-1 cells was not due to a general increase in the phagocytic activity of these cells.

## Discussion

Bacteria, especially those that establish chronic infections in their hosts, have developed a wide range of strategies to evade the innate and adaptive immune response. One of such strategies is the release of OMVs, which in some cases have been shown to exert immunomodulatory effects that favor the establishment of the infection [17]–[19]. *Brucella* spp. are intracellular bacteria that establish chronic infections in their hosts and for which several strategies of immune evasion have been described [28]. Interestingly, it has been reported that *Brucella* species produce OMVs [20], [21], [29], thus raising the possibility that these vesicles may also mediate immunomodulatory effects that favor bacterial invasion and survival in the host. In the present study we characterized the interaction of *B. abortus* OMVs with phagocytic and non-phagocytic human cells and evaluated whether such interaction results in immunomodulatory effects.

The OMVs obtained in the present study from *B. abortus* resembled those described previously for other *Brucella* species [20], with diameters ranging from 30 to 178 nm. Confocal microscopy analyses as well as flow cytometry studies revealed that some OMVs adhere to the membrane of epithelial or monocytic cells while others are detected inside the cells. The internalization of OMVs into mammalian cells *in vitro* is in line with similar findings reported for OMVs from other bacteria, such as enterotoxigenic *Escherichia coli* and *Porphyromonas gingivalis* [14], [15], and strongly suggests that OMVs may act as carriers of *Brucella* antigens not only to the surface of cells but also to intracellular localizations. This delivery system may allow the interaction of *Brucella* antigens with cellular receptors before the contact of the whole bacterium with host cells, and such interaction may underlie the different cellular effects of OMVs detected during this study.

Mammalian cells use different endocytic pathways to internalize macromolecules and/or particles, including clathrin-mediated endocytosis, invagination of cholesterol-enriched microdomains known as lipid rafts or caveolae, and formation of large F-actin coated vacuoles (phagocytosis and macropinocytosis) [25]. Monodansylcadaverine (MDC) appears to be a relatively specific blocker of clathrin-mediated internalization, whereas Fillipin III, a cholesterol-binding molecule, blocks internalization through lipid rafts. Cytochalasin D blocks actin polymerization and has been shown to block membrane ruffling and to inhibit macropinocytosis and phagocytosis under various experimental conditions. Nevertheless, pharmacological inhibition of actin polymerization has been shown to block also endocytosis via clathrin-coated pits and caveolae, suggesting that cytochalasin D should be considered a global inhibitor of all internalization pathways [25]. In the present study internalization of *B. abortus* OMVs by THP-1 monocytes was reduced by preincubation of cells with Cytochalasin D or with MDC, but it was not affected by Filipin III, thus suggesting that internalization depends, at least in part, on clathrin-mediated endocytosis. There are few reports on the mechanisms of internalization for OMVs of other bacteria. Clathrin-mediated uptake has been reported for OMVs of *Helicobacter pylori*, whereas caveolae-mediated uptake has been reported for OMVs of *Haemophilus influenzae* and *Moraxella catarrhalis* [24], [30], [31]. While the entry of OMVs from *B. abortus* to human cells may not necessarily follow the same pathways that the entry of the whole bacterium, it is interesting to note that clathrin-mediated endocytosis of *B. abortus* has been reported in Vero cells [32]. Other studies have shown the involvement of lipid rafts in the entry of *Brucella suis* into murine macrophages [33], but similar studies in human monocytes are lacking.

Importantly, the association of *Brucella* OMVs with host cells, and in particular the internalization of such OMVs, suggests that these vesicles may act as a carrier system to deliver *Brucella* antigens, including virulence factors, to host cells. As shown previously for other pathogens, the interaction of OMVs with host cells may induce either the stimulation or the downregulation of immune responses [5], [6]. OMVs contain not only LPS but also porins and other important activators of innate immunity [6]. It has been shown that OMVs from *Salmonella enterica* serovar Typhimurium are potent stimulators of proinflammatory cytokine secretion and immune cell activation [34], and OMVs from *H. pylori* and *P. aeruginosa* have been shown to elicit a potent IL-8 response [35], [36]. In the case of *P. aeruginosa*, both LPS and protein components of OMVs have been implicated in the innate immune response elicited [37]. In contrast, OMVs from different bacteria have been shown to downregulate immune responses. For example, *P. gingivalis* OMVs mediate CD14 degradation in human macrophages [17] and inhibition of the IFN-gamma-induced synthesis of MHC II molecules in endothelial cells [18]. OMVs from *Actinobacillus actinomycetemcomitans* contain a leukotoxin that kills human polymorphonuclear leukocytes and monocytes [19]. The function of CD4+ T lymphocytes is suppressed by OMVs from *Neisseria meningitidis* [38].

As mentioned, OMVs from *B. abortus* have a complex composition that includes LPS, outer membrane proteins (OMPs), periplasmic proteins and other components [21]. While some OMPs from *Brucella* have been shown to induce proinflammatory responses in different cell types [39], [40], Omp25 was reported to inhibit TNF-α secretion in human monocytes [23]. Since all these OMPs have been detected in OMVs from *B. abortus* [21], both proinflammatory and antiinflammatory stimuli may result from the interaction of these OMVs with human cells, with the final outcome probably resulting from the balance between both types of stimuli. In the present study, preincubation with *Brucella* OMVs inhibited the TNF-α response and the IL-8 response of THP-1 cells to stimulation with *E. coli* LPS (TLR4 agonist), Pam3Cys (TLR2 agonist) and flagellin (TLR5 agonist). Similarly, preincubation of THP-1 cells with OMVs resulted in a significant inhibition of the cytokine response (TNF-α and IL-8) to *B. abortus* infection. Therefore, these results indicate that the previous interaction of OMVs with human monocytes downregulates the production of proinflammatory cytokines by these cells in response to a subsequent *B. abortus* infection, possibly contributing to the immune evasion mechanisms of these bacteria. These phenomena may well take place *in vivo*, as in this study about 10^10^ CFU of *B. abortus* were required to produce 1 µg of OMVs, whereas the bacterial load in some tissues of infected animals has been reported to reach up to 10^13^ CFU per gram [41], [42].

OMVs from *B. abortus* contain several OMPs [21], which may exert different immunomodulatory effects. In particular, purified recombinant Omp16 or Omp19 have been shown to reduce the IFN-γ-induced expression of MHC-II molecules in human monocytes [22]. In the present study the treatment with different doses of OMVs resulted in a significant reduction of the IFN-γ-induced MHC-II expression in THP-1 monocytes as compared to cells only treated with IFN-γ. The expression of MHC-II molecules on the surface of *Brucella*-infected monocytes and macrophages is of utmost importance for the control of *Brucella* infection as these molecules present peptides derived from *Brucella* antigens to specific CD4+ T cells, including Th1 cells. The latter become activated as a result of antigenic recognition and produce IFN-γ, which in turn activates several antibacterial mechanisms in the infected cell, including the production of nitric oxide. Several studies have shown that the production of IFN-γ by activated Th1 cells is essential for the control of *Brucella* infection [43], [44]. Therefore, the downregulation of MHC-II expression mediated by OMVs may constitute a strategy for attenuating Th1-mediated immune responses against *Brucella*-infected cells.

As mentioned, OMVs contain a complex mixture of outer membrane and periplasmic bacterial antigens, some of which may act as stimulants of innate immune responses. Apart from the cytokine responses mentioned above, one of such innate responses may be the increased expression of molecules that contribute to the adhesion of immune cells to the endothelium and their subsequent migration towards the focus of infection. One of such molecules, the intercellular adhesion molecule-1 (ICAM-1) is expressed on both phagocytes and endothelial cells, and its surface expression is induced by different stimuli, including microbial antigens and proinflammatory cytokines [45], [46]. We found that OMVs induced a dose-dependent increase of ICAM-1 expression on human monocytes (THP-1 cells). Moreover, using a functional study we demonstrated that interaction of monocytes with *Brucella* OMVs results in an increased adhesion of these cells to the endothelium, and that OMVs can also stimulate the adhesive properties of endothelial cells. Overall, these results suggest that *Brucella* OMVs stimulate the expression of adhesion molecules on the surface of both monocytes and endothelial cells, thus favoring adhesive interactions between these cell types. Further studies are needed to assess the potential consequences of such increased adhesion during *in vivo* infections.

As discussed above some of the effects exerted by *Brucella* OMVs, such as TNF-α inhibition and MHC-II downregulation, may favor the persistence of the bacterium in macrophages. We wondered whether the previous or simultaneous interaction of OMVs with target cells may also influence the internalization of *Brucella* by such cells. We found that both preincubation and coincubation with OMVs results in an increased number of internalized bacteria in THP-1 cells. These results suggest that OMVs released by *B. abortus* before or during the interaction with macrophages produce a significant increase in *Brucella* internalization by such cells. Given the ability of *Brucella* to survive within monocytes/macrophages and to establish its replication niche in these cells [47], [48] this increased uptake of the bacterium may favor the establishment of persistent *Brucella* infections within the host. Similar to our findings with *Brucella* OMVs, it has been reported that OMVs from *Actinobacillus actinomycetemcomitans* enhance the adherence of this bacterium to oral epithelial cells [49]. The mechanism involved in the increased uptake of *Brucella* is unknown, but it can be speculated that the recognition of OMVs by macrophages triggers the activation of these cells, leading to an increased phagocytic activity and/or the increased surface expression of a preformed molecule that serves as a receptor for *Brucella*. The first possibility was ruled out by experiments in which the pretreatment or concomitant treatment with OMVs from *Brucella* did not modify the phagocytic activity of macrophages for latex beads. These results seem to favor the second hypothesis about an OMV-triggered increase in the surface expression of a cellular receptor for *Brucella*.

In summary, the present study shows that OMVs from *B. abortus* exert various effects on human monocytes, including a reduced ability of these cells to secrete proinflammatory cytokines in response to the bacterium, a diminished capacity to express MHC-II molecules in response to IFN-γ stimulation, an increased expression of adhesion molecules, and an enhanced capacity to internalize *B. abortus*. Therefore, OMVs may on the one hand promote the internalization of *B. abortus* by monocytes, but on the other hand may also induce a downregulation of the innate immune response of these cells to *Brucella*. These effects may act in concert to favor the entry and persistence of the bacterium within host cells, thus contributing to the chronic nature of brucellosis.
